# Transfer learning-based hybrid VGG16-machine learning approach for heart disease detection with explainable artificial intelligence

**DOI:** 10.3389/frai.2025.1504281

**Published:** 2025-02-25

**Authors:** Eshetie Gizachew Addisu, Tahayu Gizachew Yirga, Hailu Gizachew Yirga, Alemu Demeke Yehuala

**Affiliations:** ^1^Department of Information Systems, College Informatics, University of Gondar, Gondar, Ethiopia; ^2^Department of Computer Science, College of Natural and Computational Science, Mekdela Amba University, Tulu Awuliya, Ethiopia; ^3^Department of Computer Science, College of Informatics, University of Gondar, Gondar, Ethiopia; ^4^Department of Surgery, College of Medicine and Health Science, University of Gondar, Gondar, Ethiopia

**Keywords:** machine learning, deep learning, heart disease, artificial intelligence explainability, feature extraction, VGG16-random forest, CTGAN

## Abstract

Heart disease is a leading cause of mortality worldwide, making accurate early detection essential for effective treatment and management. This study introduces a novel hybrid machine-learning approach that combines transfer learning using the VGG16 convolutional neural network (CNN) with various machine-learning classifiers for heart disease detection. A conditional tabular generative adversarial network (CTGAN) was employed to generate synthetic data samples from actual datasets; these were evaluated using statistical metrics, correlation analysis, and domain expert assessments to ensure the quality of the synthetic datasets. The dataset comprises tabular data with 13 features, which are reshaped into an image-like format and resized to 224x224x3 to meet the input requirements of the VGG16 model. Feature extraction is performed using VGG16, and the extracted features are then fused with the original tabular data. This combined feature set is then used to train various machine learning models, including Support Vector Machines (SVM), Gradient Boosting, Random Forest, Logistic Regression, K-nearest neighbors (KNN), and Decision Trees. Among these models, the VGG16-Random Forest hybrid achieved notable results across all evaluation metrics, including 92% accuracy, 91.3% precision, 92.2% recall, 91.82% specificity, 92.2% sensitivity, and 91.75% F1-score. The hybrid models were also evaluated using unseen datasets to assess the generalizability of the proposed approaches, with the VGG16-Random Forest combination showing relatively promising results. Additionally, explainability is integrated into the model using SHAP values, providing insights into the contribution of each feature to the model’s predictions. This hybrid VGG16-ML approach demonstrates the potential for highly accurate and interpretable heart disease detection, offering valuable support in clinical decision-making processes.

## 1 Introduction

The term “heart disease” has become increasingly common, with the World Heart Federation (WHF, 2021) projecting that the number of related deaths will rise to 22.5 million by 2025. These alarming statistics highlight the urgent need for scientific research and medical breakthroughs aimed at preventing and mitigating the impact of cardiovascular diseases globally. Key risk factors contributing to the development of cardiovascular diseases include high blood pressure, excess body weight and obesity, abnormal lipid profiles, irregular glucose levels or diabetes, tobacco use or smoking habits, physical inactivity or sedentary lifestyles, alcohol consumption, and high cholesterol levels.

The most crucial organ in the human body is the heart, making comprehensive early evaluation and accurate prognosis of heart disease vital. To address this, conducting thorough research on the subject is essential. The primary reason patients succumb to these illnesses is the typically late prediction of the conditions. Therefore, it is essential to develop reliable methods for early heart disease prediction (Chudhey et al., 2022).

Early diagnosis of heart disease is crucial in the medical field, as it can save lives and prevent severe consequences (Mulyani et al., 2024). The complexity of heart diseases, coupled with the financial burden of healthcare, highlights the need for effective digital solutions to enhance cardiovascular health and wellness. Deploying artificial intelligence (AI)-based, task-specific solutions equipped with explainability mechanisms can help mitigate the shortcomings and biases of the existing healthcare system.

In the current medical landscape, artificial intelligence-aided frameworks can help diagnose individuals with heart diseases, enhancing the likelihood of favorable health outcomes. Numerous studies on emerging AI techniques have focused on the classification and prognosis of heart diseases (Shrivastava et al., 2023). While several machine-learning approaches have been deployed to identify heart diseases (Khan Mamun and Elfouly, 2023), the majority of these approaches demonstrate low accuracy, lack the integration of optimal feature extraction techniques from convolutional neural networks (CNN) into machine-learning predictive models, and fail to provide transparency in the reasoning behind their predictions, which is crucial for understanding and validating the predicted outcomes. Hybrid approaches that combine the strengths of deep learning and machine learning are increasingly promising for early heart disease prediction. This study presents a hybrid deep learning and machine learning technique with an explainable paradigm-based approach for heart disease identification aimed at addressing existing shortcomings and challenges. The proposed model leverages the advanced feature extraction capability of the Visual Geometry Group (VGG16) with predictive machine learning algorithms, utilizing a transfer learning mechanism to enhance the accuracy of heart disease diagnosis.

VGG16 is a pre-trained, specific type of convolutional neural network architecture. Its capacity to extract significant features from data has made it more well-known in recent years (Bakar et al., 2023). The CNN-based VGG16 is used for improved performance since it is trained on millions of ImageNet datasets. CNNs are able to extract pertinent features from raw data automatically without the need for human feature engineering from datasets such as images (Mulyani et al., 2024). This increases the accuracy of disease classification by enabling CNNs to recognize intricate patterns and relationships in the data.

Machine learning (ML) is the application of artificial intelligence that deals with creating a model that can learn and predict outcomes based on historical data and experiences by training and testing with features of these datasets. It has been shown that machine learning methods are very useful predictors (Subramani et al., 2023). ML approaches leverage their potential classification performance in different domains, including heart disease detection. The accuracy of ML models is determined by the size of datasets used for training; the more datasets used, the more accurate the machine learning model will be. Transfer learning is a machine learning technique wherein insights from one task or dataset are applied to enhance model performance on a different or related dataset, and it is an ideal solution when the size of the dataset is an issue. Combining the sophisticated feature extraction capabilities of a CNN-based pre-trained model with the potential classification performances of ML techniques by applying transfer learning mechanisms for heart disease detection is a novel approach.

Therefore, the main aim of this study was to design a transfer learning-based hybrid VGG16-ML model that can leverage the strengths of powerful deep learning feature extraction capability, machine learning’s classical classification, and interpretability performance to provide a robust solution for heart disease detection. The proposed hybrid predictive model can explain how deep learning and tabular features contribute to the model’s decision-making process by integrating the so-called artificial intelligence explainability(XAI) methods. XAI consists of tools and architectures designed to make ML classifiers more transparent and provide a similar context for the reasoning behind a particular prediction. These resources are frequently employed to transparently illustrate the hidden black-box patterns of artificial intelligence. Building trust and confidence to accept forecasts from a decision support system requires the use of XAI approaches. SHapley Additive exPlanations (SHAP) were integrated into this study. SHAP is a mathematical tool for explaining machine learning models that allow one to compute the contribution of each feature to the prediction using SHAP values (Lundberg and Lee, 2017). Consequently, SHAP was utilized in this study to clarify how the hybrid heart disease predictive model reaches a decision.

The study aims to detect heart disease levels systematically and use an efficient transfer learning-based hybridized model by integrating the strengths of both deep learning and machine learning approaches. The goals intended to be achieved through this study were listed as follows: (i) to efficiently preprocess and transform the tabular datasets into images to extract features. (ii) To propose a hybrid model comprising VGG16 with a Support Vector Machine (SVM) to classify heart disease. (iii) To propose a hybrid model comprising VGG16 with Random Forest (RF) for classifying heart disease types. (iv) To propose a hybrid model comprising VGG16 with K-Nearest Neighbour (KNN) for classifying heart disease types. (v) To propose a hybrid model comprising VGG16 with Logistic Regression (LR) to classify heart disease types. (vi) To propose a hybrid model comprising VGG16 with Gradient Boosting (GB) to classify heart disease types. (vii) To propose a hybrid model comprising VGG16 with a Decision Tree (DT) for classifying heart disease types. (viii) To employ hyper-parameter tuning to optimize the performance of the proposed various hybrid models. (ix) To analyze the model performance based on various metrics such as accuracy, precision, recall, F1-score, sensitivity, specificity, and computational time in seconds. (viii) To employ XAI techniques on the proposed hybrid models. (ix) To compare and analyze the performance of proposed models with standalone supervised machine learning classifiers without VGG16 feature extractors. (x) Various evaluation mechanisms should be employed to evaluate the effectiveness of this hybrid approach.

## 2 Related studies

In the medical domain, specifically in the field of heart disease, researchers have carried out many studies by incorporating artificial intelligence techniques, including deep neural networks and machine learning techniques. Hence, different model development techniques, datasets, and feature engineering methods have been used, resulting in varying outcomes. Some of the studies related to the proposed methods have been highlighted below.

Anbazhagan et al. (2024) proposed a hybrid approach that combines machine learning and deep learning techniques for early heart disease prediction by integrating the XGBoost-LSTM model to comply with the interpretability of XGBoost with the temporal modeling capabilities of LSTM models to analyze structured and unstructured data. The hybrid approach was proposed to carry out heart disease prediction by initially applying XGBoost to generate predictions and capture intricate connections as feature extractions from the structured data, followed by recording temporal relationships from time series data relevant to the final prediction of heart disease using LSTM. That study utilized data from the UCI machine learning repository, feature engineering guided by domain expertise, and transfer learning. This hybrid early heart disease prediction approach achieves superior results over standalone machine learning models in accuracy, precision, sensitivity, and F1-score evaluation metrics.

A hybrid model of CNN-bidirectional long-short-term memory(CNN-BiLSTM) was investigated on tabular datasets collected from Cleveland UCI datasets for heart disease, aiming at building an efficient heart disease prediction model(Shrivastava et al., 2023). Data preprocessing techniques were employed over publicly available datasets to handle the data imbalance and missing data value concerns. In addition, an extra tree classifier was used to select relevant features and eliminate the least important ones from publicly available datasets. The CNN model was used to extract features, and finally, the classification process was performed by the Bi-LSTM model. Even though the dataset is small, promising results on accuracy, precision, recall, and F1-score were achieved during an experiment on the datasets.

An ensemble learning-based hybrid deep learning model for early heart disease detection was developed in Egypt by integrating two optimized and pre-trained models with a support vector machine, namely CNN with Long-Short Term Memory (LSTM) and CNN with Gated Recurrent Unit (GRU), to enhance heart disease prediction using simple data and symptoms (Almulihi et al., 2022). This ensemble learning model uses recursive feature elimination for feature selection purposes, and it is compared with some machine learning models (Almulihi et al., 2022). The result from the proposed model achieved better performance due to the optimization techniques used for the deep learning and machine learning models (Almulihi et al., 2022).

Mandava et al. (2023) investigated inclusive machine learning and deep learning methods to predict cardiovascular disease in Bangladesh with the main intention of helping medical practitioners and improving the accuracy of prediction models to lower severe risks from heart disease. In that study, heart disease predictive models have been created and analyzed using various machine learning approaches with an accuracy of 96.7%, indicating that deep learning algorithms can help with the recognition, categorization, and quantification of patterns found in medical imaging, which can enhance patient assessment and diagnosis by considering past medical records and evaluation patterns (Mandava et al., 2023).

Bharti et al. (2021) explored the application of machine learning and deep learning in a combined fashion by integrating with a multimedia technology like a mobile device for improved heart disease prediction and making a comparison and analysis by UCI machine learning heart disease datasets. In their study, mainly machine learning and deep learning techniques are compared with datasets with outlier detection and preprocessing and without outlier detection and preprocessing. Deep learning algorithms with isolation forest techniques for outlier detection and lasso algorithms for feature selection achieve promising accuracy (Bharti et al., 2021).

A study conducted in Nepal used ensemble learning to predict potential heart risk, employing five supervised machine learning algorithms combined with publicly available UCI heart disease datasets to build an ensemble model (Adhikari and Shakya, 2022). The authors concluded that the ensemble model achieves better accuracy compared to individual supervised machine learning models. Specifically, the base ensemble model reached an accuracy of 96.43%, while the voting-based ensemble model achieved an accuracy of 96.10%.

Mehmood et al. (2020) investigated early heart failure predictive methods to help patients and medical practitioners using a convolutional neural network and named it CardioHelp. The aim of the authors of the study was to exploit a convolutional neural network for temporal data modeling on heart disease datasets to predict heart failure, and the result achieved outperformed currently existing methods with an accuracy result of 97% (Mehmood et al., 2020).

Hossain et al. (2023) conducted a study to process sequential data and identify dependencies and patterns across time, using an LSTM network in conjunction with a CNN aimed to extract pertinent features from the input data. This work sheds light on how a hybrid deep learning model, in conjunction with explainable artificial intelligence and feature engineering, may enhance the precision and comprehensibility of heart disease prediction (Hossain et al., 2023). The resulting model was tested using a publicly accessible dataset, and the suggested CNN-LSTM outperformed the state-of-the-art models in identifying people with heart disease with a high accuracy of 73.52 and 74.15%, with and without feature engineering techniques, respectively. This study explores the role of explainable AI in knowing the most relevant features in heart disease detection and the power of hybrid deep learning models for enhanced early heart disease detection (Hossain et al., 2023).

Uma Maheswari et al. (2021) investigated the performance of three machine learning techniques on heart disease prediction, namely random forest, decision tree, and hybrid of decision tree and random forest on Cleveland heart disease datasets. The result gained from the experiment conducted by that study shows that the hybrid model outperforms the random forest and decision tree models with an accuracy result of 88.7% (Uma Maheswari et al., 2021).

Mohan et al. (2019) proposed an innovative idea of applying machine learning techniques to identify significant features, resulting in an enhanced accuracy of heart disease prediction. Thus, various machine learning classification techniques and features were combined in different ways to introduce a heart disease predictive model with an enhanced accuracy performance of 88.7% by hybridizing random forest with a linear model (Mohan et al., 2019).

In a study by Shah et al. (2020), various supervised machine learning algorithms such as Naïve Bayes, decision tree, K-nearest neighbor, and random forest have been examined using publicly available Cleveland heart disease datasets to present a heart disease predictive model with various attributes. The primary objective of this study was to estimate the likelihood of patients developing heart disease, with the K-nearest neighbors algorithm achieving better accuracy results.

Louridi et al. (2021) conducted a study to develop a machine learning-driven intelligent medical system designed to assist in assessing a patient’s cardiac health and support physicians in accurately diagnosing cardiovascular disorders. The author addressed the issues of imbalanced and missing data in the publicly accessible Framingham and UCI heart disease datasets by employing various data processing techniques. They selected the optimal method for predicting cardiovascular disease using machine learning. The efficacy of the proposed system was evaluated using various metrics, including accuracy, sensitivity, F-measure, and precision, demonstrating that the suggested technique significantly outperforms alternative models.

Machine learning models based on related parameters have been built for heart disease prediction, with 14 features having a relation with cardiovascular disease (Singh and Virk, 2023). In this research work, UCI heart disease datasets and supervised machine learning algorithms such as random forest, support vector machine, Naïve Bayes, and decision tree were used to develop the predictive model. Standard machine learning methods were used to identify the correlation between different attributes on the datasets, which has a significant role in the probability of predicting heart disease. The random forest algorithm gives relatively better accuracy and less computation time for heart disease prediction, as shown in the experimental result.

In Bangladesh, a study was conducted to build heart disease predictive models with local datasets of 564 instances and 18 attributes using machine learning algorithms. The main objective was identifying the most relevant features in heart disease prediction, and the model was trained with supervised machine learning techniques, including K-nearest neighbor, decision tree, Naïve Bayes, support vector machine, and logistic regression. According to the result of the study, the support vector machine outperforms the other algorithms, and an accuracy result of 91% was achieved (Shaw and Patidar, 2023).

A heart disease prediction model based on an ensemble technique using extra tree classifier methods for feature selection targeted at selecting the most relevant feature combination for enhanced early heart disease prediction was proposed. To build the proposed model, K-Nearest Neighbour, support vector machine, Naïve Bayes, decision tree, logistic regression, and Vote classifiers are examined on Cleveland and Statlog datasets by three different scenarios. Hence, the classifiers were tested with all the main 13 features of the dataset, with nine feature combinations and six feature combinations. In the end, the relatively best F1 score and accuracy result was achieved from the Vote classifier using nine and six feature combinations (Baranidharan et al., 2019).

In conclusion, various studies have been conducted for early heart disease prediction using machine learning and deep learning techniques. Promising performances were attained from multiple approaches. The majority of researchers highlight and recommend the utilization of the best capabilities of machine learning and deep learning techniques, appropriate feature extraction, and hyperparameter tuning mechanisms to create a hybridized model for enhanced early heart disease predictions. Moreover, several studies have been conducted to build heart disease predictive models with rigorous feature engineering mechanisms from publicly available heart disease datasets, but no study was focused on integrating the feature extraction capability of deep learning methods and classification capabilities of machine learning techniques to build a transfer learning-based hybrid model for improved robust heart disease detection. Thus, this study aims to build a hybrid model of deep learning, and machine learning approaches for heart disease prediction using explainable artificial intelligence techniques to show the transparency of how the proposed models reach the prediction of heart disease decision-making processes.

## 3 Materials and methods

### 3.1 Materials

The datasets for this study were used from the publicly available Kaggle data repository, comprising 1,025 and an additional 1,100 synthetic data generated from the original data samples, and 14 features were used for this study, as described in Table 1. The original dataset (1,025) consists of four databases, namely Cleveland, Hungary, Switzerland, and Long Beach V. It contains 76 attributes, including the predicted attribute, but all published experiments refer to using a subset of 14 of them, including the target column. Synthetic data could be generated in different domains, including in medicine, similar to synthesizing breast cancer datasets if the dataset size is limited, and a conditional tabular generative adversarial network (CTGAN) is an ideal solution to generate additional tabular datasets for this study (Inan et al., 2023). The dataset for this study includes 2,125 total 1,176 instances of suffering from heart disease and 1,049 instances of healthy heart datasets. The focus on generating and using synthetic data addresses the issues with small datasets and opens up possibilities for more extensive study in the area of heart attack prediction(Singh and Kirar, 2024).

**Table 1:** Description of heart disease datasets.

Attributes	Description	Type
Age	Age of the patient in a year.	Numerical
Sex	Gender of the patient. Male:0 and female:1	Nominal
cp	Types of chest pain. 0: typical angina 1: atypical angina 2: non-anginal pain 3: asymptomatic	Nominal
trestbps	Blood pressure at resting mode in mm/HG.	Numerical
chol	Serum cholesterol in mg/dl.	Numerical
fbs	Blood sugar levels on fasting greater than 120 mg/dL. 1: for satisfying and 0: failed to satisfy the condition, respectively	Nominal
restecg	Electro diagram results at rest: 0: normal 1: having ST-T wave abnormality 2:showing left ventricular hypertrophy	Nominal
thalach	Maximum heart rate	Numerical
exang	Angina induced by exercise: 0:no, 1:yes	Nominal
oldpeak	Exercise-induced ST depression in relation to the state of rest	Numerical
slope	ST segment in terms of slope during exercise. 0: up sloping, 1:flat, 2:down sloping	Nominal
ca	The number of major vessels(0–3)	Nominal
thal	Thalassemia: 0:NULL, 1:normal 2:fixed defect, 3:reverseible defect	Nominal
taregt	Results of the instances provide: 1: patient suffering heart disease, 0: patient is normal	

### 3.2 Methodology

**Figure 1:**
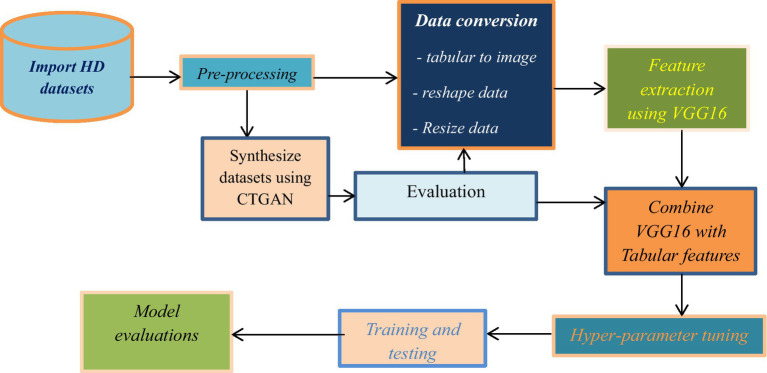
Workflow of the study.

This study achieved its overall goal through the progressive application of the following methodologies. To realize the proposed hybrid heart disease predictive model, as depicted in Figure 1, these series of activities have been used as a general methodology for the study.

#### 3.2.1 Data preprocessing

##### 3.2.1.1 Data preparation

Data preprocessing is a crucial initial step in machine learning before analyzing data or developing a model. Data preprocessing was carefully performed to address issues such as missing values, imbalanced distributions, and outliers, ensuring a high-quality dataset for the proposed model.

##### 3.2.1.2 Data normalization

There are well-known data normalization techniques such as min-max normalization, decimal scaling, unit vector normalization, Z-score normalization, and log transformation. In this study, min-max normalization was selected due to its capability to carry out linear modifications on the original data to equalize value comparisons between data before and after the process (Ahsan et al., 2023). We used the min-max normalization technique to scale our input data into ranges of 0 to 1. Min-Max normalization transforms the original input data in to suitable format to improve data consistency and this method can be expressed in the formula indicated on Equation 1 (Henderi, 2021):


(1) $$X{\mathrm{new}}_{=}\frac{X-\min\left(X\right)}{\max\left(X\right)-\min\left(X\right)}$$

Where *X*new = The new value generated by normalizing the original.

*X* = The original value of data.

*max(X)* = The maximum value in the original dataset.

*min(X)* = The minimum value in the original datasets.

##### 3.2.1.3 Data conversion

Data conversion involves the process of transforming tabular data into images, which are vital input formats for VGG16. This process of data conversion improves the effectiveness of the VGG16 model in extracting features from data that originally did not have spatial correlation by converting them into compacted grayscale images. The results achieved by Jain et al. (2022), which investigated the application of a novel approach to adopt deep learning methods on small tabular datasets using transfer learning, inspired us to conduct this study. In their study, different techniques, including image generator for tabular data (IGTD), representation of features as images with neighborhood dependencies (REFINED), and supervised tabular machine learning (SuperTML), were incorporated to convert tabular datasets into two-dimensional image representations in order to prepare image inputs for image classification tasks. The classification result achieved in this way by using a transfer learning approach on small datasets outperforms the classification result achieved by the conventional machine learning classifiers. In a study by Damri et al. (2024), the results of experiments highlighted that transforming tabular data into an image-like format for image classification using deep learning approaches outperforms conventional machine learning classifiers on structured data. Encouraged by these findings to leverage CNN-based techniques such as VGG16 for feature extraction from originally tabular datasets, this study undertook the transformation of tabular datasets into images. The transformation involved specific preprocessing steps aimed at integrating the efficiency of deep learning approaches while addressing the challenges associated with small and tabular datasets.

- Reshaping the data: This process involves a set of activities required to transform the original tabular dataset into two-dimensional image-like formats in order to produce relevant inputs for the VGG16 pre-trained models. To do this, we incorporate the following three core steps to create an image-like format that retains the spatial relation and interaction of features within the dataset. First, to group features together based on their similarity, hierarchical clustering was applied to the correlation matrix values (revealing how the features from the dataset are related to each other). For this purpose, a pairwise correlation was computed between all features using a Pearson correlation, and to perform the hierarchical clustering, the distance matrix was computed from the correlation matrix. Finally, the order of features was extracted from the resulting clustering. Second, features of the datasets were reordered based on resulting ordered feature indices from the generated hierarchical clustering. This procedure allows us to retain general spatial relations and interactions represented by the features of the datasets. This reordering of features’ positions on the dataset helps us to represent similar features close together in the formulation of proposed image-like formats. Third, converting the dataset into a two-dimensional image format was the next task. To do this, the reordered feature values of each sample from the dataset were reshaped into a 4 × 4 grid (each row of the tabular dataset is mapped into the two-dimensional matrix or format, and the respective normalized feature value represents the color intensity). In doing that, since the number of features does not fit perfectly in the grid, padding was necessary in order to fill the fourth row’s last three columns with zero values (zero padding).
- Resize the image and apply color channels: After reshaping, we resized the 4 × 4 image into a 224 × 224 format with three channels (224 × 224 × 3) to match the input requirements for the VGG16 model for better implementation of transfer learning. During this resizing, we retained the original pixel intensity values through interpolation, preserving the grayscale representation of each feature. Finally, the image transformed from the tabular datasets, which preserved the spatial relation between features through a hierarchical clustering process, was produced to be used as valid input for VGG16 feature extraction processes.

#### 3.2.2 The proposed model development

##### 3.2.2.1 Feature extraction

The heart disease datasets converted into the images in the previous steps were fed into the VGG16 pre-trained model, whose top layer was frozen to extract general features through convolutions without training from scratch. The VGG16 model’s convolution operation applies filters to the input image to extract essential features. Filters are capable of extracting specific patterns such as textures, edges, and other complex shapes while the network goes deeper. The output of the VGG16’s final convolutional layer was used as a feature vector learned from the input images as high-level representations from the network.

##### 3.2.2.2 Data fusion

Data fusion involves the process of combining features extracted from the VGG16 model with the original tabular features. Such approaches in building hybrid machine learning predictive models allow us to leverage both the deep learning capabilities of VGG16 and the structured information in tabular data to enhance prediction accuracy. Finally, the combined enriched feature set representation incorporates both structured, manually recorded clinical data and the abstract, high-level patterns extracted by the VGG16 model with a more comprehensive view of each instance. Hence, the number of columns increases due to column-wise concatenation of both features for single instance representation.

##### 3.2.2.3 The proposed VGG16-machine learning models

Machine learning algorithms such as super vector machine, logistic regression, decision tree, random forest, and K-nearest neighbor were trained and tested with the combined features of both raw tabular features and VGG16-extracted features. VGG16 is basically used to extract essential features from the input data through convolutional operations. The filters in the VGG16 convolution processes capture and learn the patterns on the data inputs to identify the most relevant features (Torres et al., 2021). The output from the VGG16 convolution process is then given to machine learning component classifiers. Machine learning technologies are the ideal and most convenient techniques for disease prediction, such as in classifying heart disease (Ahmad and Polat, 2023).

Moreover, as the term “composition” suggests, the proposed hybrid VGG16 machine learning model primarily consists of two major components designed to optimize heart disease prediction. The first component is the CNN-based VGG16 pre-trained model for feature extraction. The second component is machine learning for heart disease classification. These two main components were examined over the publicly available Kaggle heart disease datasets with some adjustments and transformations as preprocessing methods. To do that, the raw tabular heart disease datasets were converted into image-like data and resized into standard input shapes, and VGG16, frozen in its top layer, was fed the adjusted image without training. Then, the outputs of VGG16 extracted from the images are combined with preprocessed tabular data versions. Finally, various machine learning approaches have been trained and analyzed over the combined datasets based on different evaluation metrics.

Furthermore, after the various machine learning approaches have been analyzed using different evaluation metrics, a hybrid VGG16-random forest model with hyperparameter tuning achieves the promising result of 92% accuracy, 91.3% precision, 92.2% recall, 91.75% F1-score, 92.2% sensitivity, and 91.82% specificity.

##### 3.2.2.4 Integrate XAI technique

As was proposed in the early stage, an explainable artificial intelligence (XAI) technique was also integrated with the proposed hybrid VGG16-ML heart disease detection model to determine the influence of each feature of datasets on the prediction of any heart disease outcomes. There are various types of XAI; we used SHapley Additive exPlanations (SHAP) for this study. It is one of the most known XAI methods in describing the machine learning model prediction results, which strengthens the knowledge of which feature was highly influential on the model’s prediction outcome and why a model made such particular predictions (Khorram et al., 2021). It is also utilized as a tool to interpret the final predictions and to gain valued insights into highly contributing features for the machine learning algorithms classification process in this study (Islam et al., 2023). Finally, the contribution of each feature on the dataset is visualized using SHAP to show their contribution to the respective target classification. Equation 2 below shows the general formula for how the SHAP value is calculated from the feature and input data point.


(2) $$\varphi i\left(f,x\right)=\underset{Z^{'}\subseteq X^{\prime }}{\sum }\frac{\left|Z^{'}\right|!\left(M-\left|Z^{'}\right|-1\right)}{M}\left[fx\left(Z^{'}\right)-fx\left(\frac{Z^{'}}{i}\right)\right]$$

Where,

ƒ = Black-box model.

*φi* = Shapley value of any feature i.

*x* = Input data point.

*ź* = the subset.

*x’* = Simplified data input.

ƒ*_x_* (ź) = with feature i.

ƒ*_x_*(ź/i) = without feature i.

**Figure 2:**
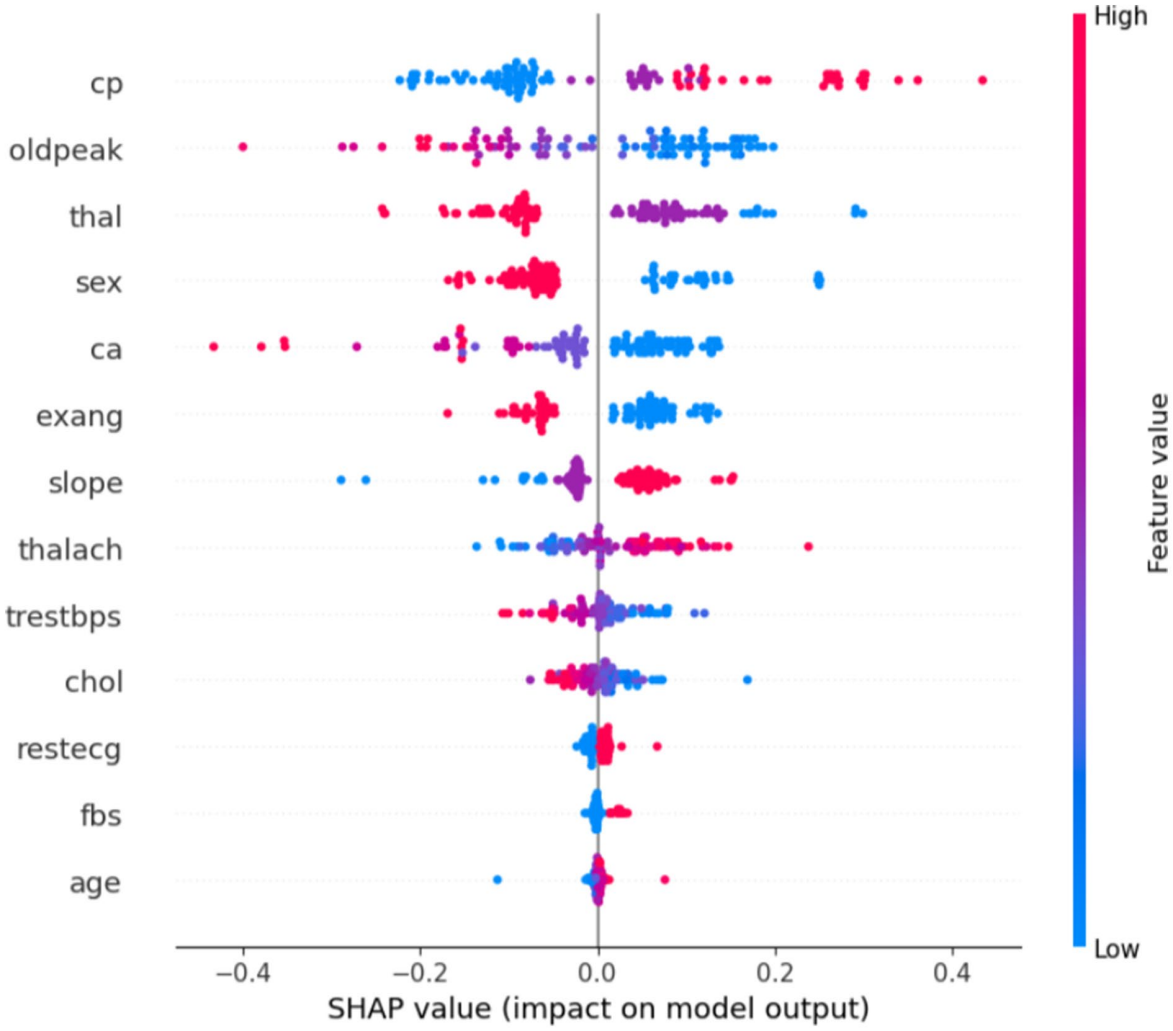
Feature impacts on predicting a case of heart disease.

Equation 2 is used to determine the impact of an individual feature by evaluating all possible subsets of features that contain that particular feature. The feature’s impact is then weighted based on the number of subsets in which it appears and averaged across all possible subsets. This results in a single SHAP value for each feature, which can be used to interpret the model’s prediction for a specific case, as it is represented in Figure 2.

The plot in Figure 2 provides insight into how various features impact the model’s prediction of heart disease versus no heart disease. The x-axis represents the SHAP value, which indicates the impact of each feature on the model’s prediction. Positive SHAP values (points on the right) push the model toward predicting heart disease, while negative SHAP values (points on the left) push the model toward predicting no heart disease. The y-axis lists the features (such as cp, oldpeak, thal, sex, and so on), ordered from the most impactful at the top to the least impactful at the bottom. The color of the points represents the actual feature values, where red indicates high feature values and blue represents low feature values. This color coding helps to visualize how each feature’s value influences the model’s decision.

In general, some features like chest pain type, oldpeak, and thalassemia show a strong impact on predicting heart disease. High values for these features are associated with the presence of heart disease. In contrast, features such as age, chol, and restecg seem to have less influence on the prediction, as their SHAP values are closer to zero.

## 4 Results

### 4.1 Datasets

The heart disease datasets from Kaggle comprising 1,025 publicly available and 1,100 synthetic data samples with 14 features were used for this study. The actual dataset consists of four databases, namely Cleveland, Hungary, Switzerland, and Long Beach V. It contains 76 attributes, including the predicted attribute, but all published experiments use a subset of 14 of them, including the target column. The “target” field refers to the presence of heart disease in the patient with two categorical values (0 and 1), where 0 represents healthy cardiac status, whereas 1 indicates signals of infected cardiac statuses. Experiments have been conducted using Jupyter Notebook (Anaconda3) in Python programming.

The generated synthetic data was then evaluated through correlation analysis, standard statistical metrics, comparison with the actual data, and expert evaluation to assess its quality, to produce realistic synthetic data resembling the distribution of the original dataset, and to ensure the similarity between the original and synthetic data. To do this, a set of preprocessing activities has been applied to the original data, such as cleaning any missing, incorrect, or outlier values using standard techniques and feature and label separation. Subsequently, CTGAN was trained on the original data using hyperparameters such as learning rate, epoch, and batch size to generate 1,100 synthetic samples.

**Figure 3:**
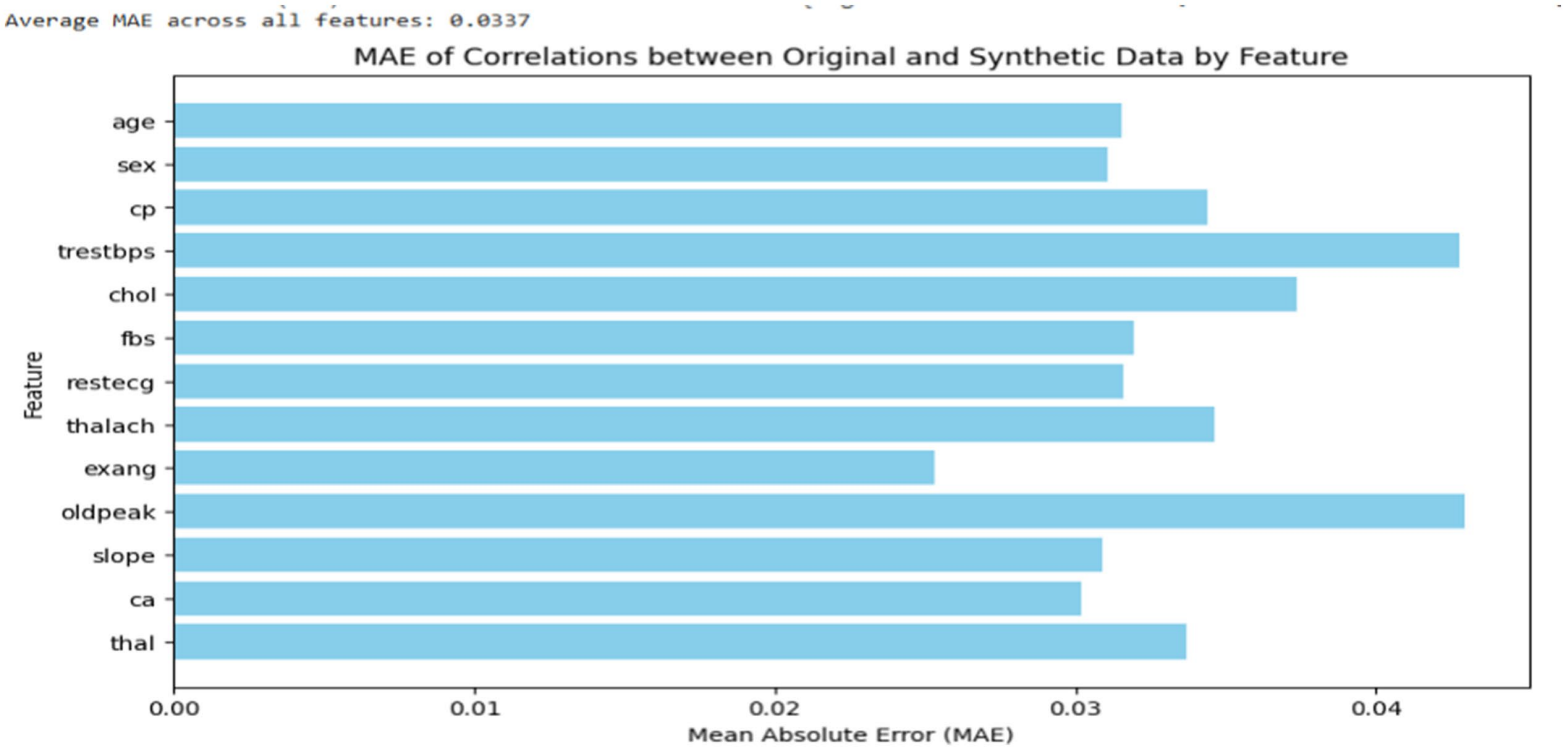
MAE of correlations by features between synthetic and actual datasets.

Subsequently, to evaluate the quality and similarity of the synthetic data with the actual datasets, correlation analysis was conducted between the synthetic data and the original dataset to evaluate how well the synthetic data retained the relationships between features. As indicated in Figure 3, the average mean absolute error (MAE) across all features was found to be 0.03, suggesting that the synthetic data was highly accurate and closely approximated the actual data. Additionally, the synthetic data were evaluated by domain experts with authors who assessed its plausibility and relevance to the application. The domain experts confirmed that the synthetic data was realistic and could be used for further analysis, confirming the CTGAN model’s ability to generate useful and credible synthetic data.

**Figure 4:**
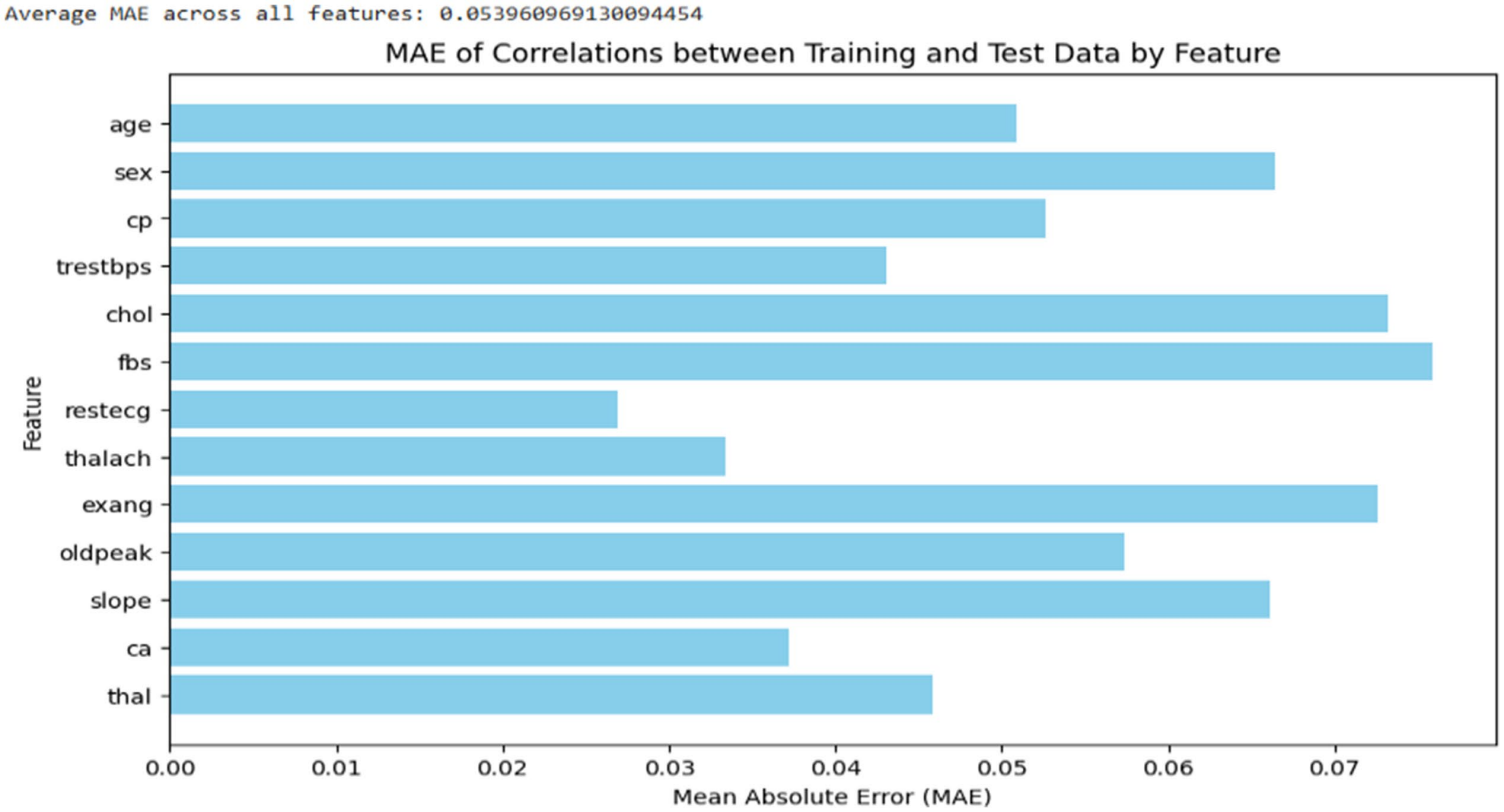
Average MAE correlation of features within the original dataset.

As shown in Figure 4, a baseline analysis was also conducted to evaluate the average MAE of feature correlations within the original data by splitting it into two sets: training and testing (50:50). The feature correlations in both the training and testing sets were visualized. The average MAE correlation between the synthetic and original data closely matched that of the baseline analysis, even showing slightly lower values. This suggests that the synthetic data generated are highly similar to the original data in terms of feature value distribution.

**Figure 5:**
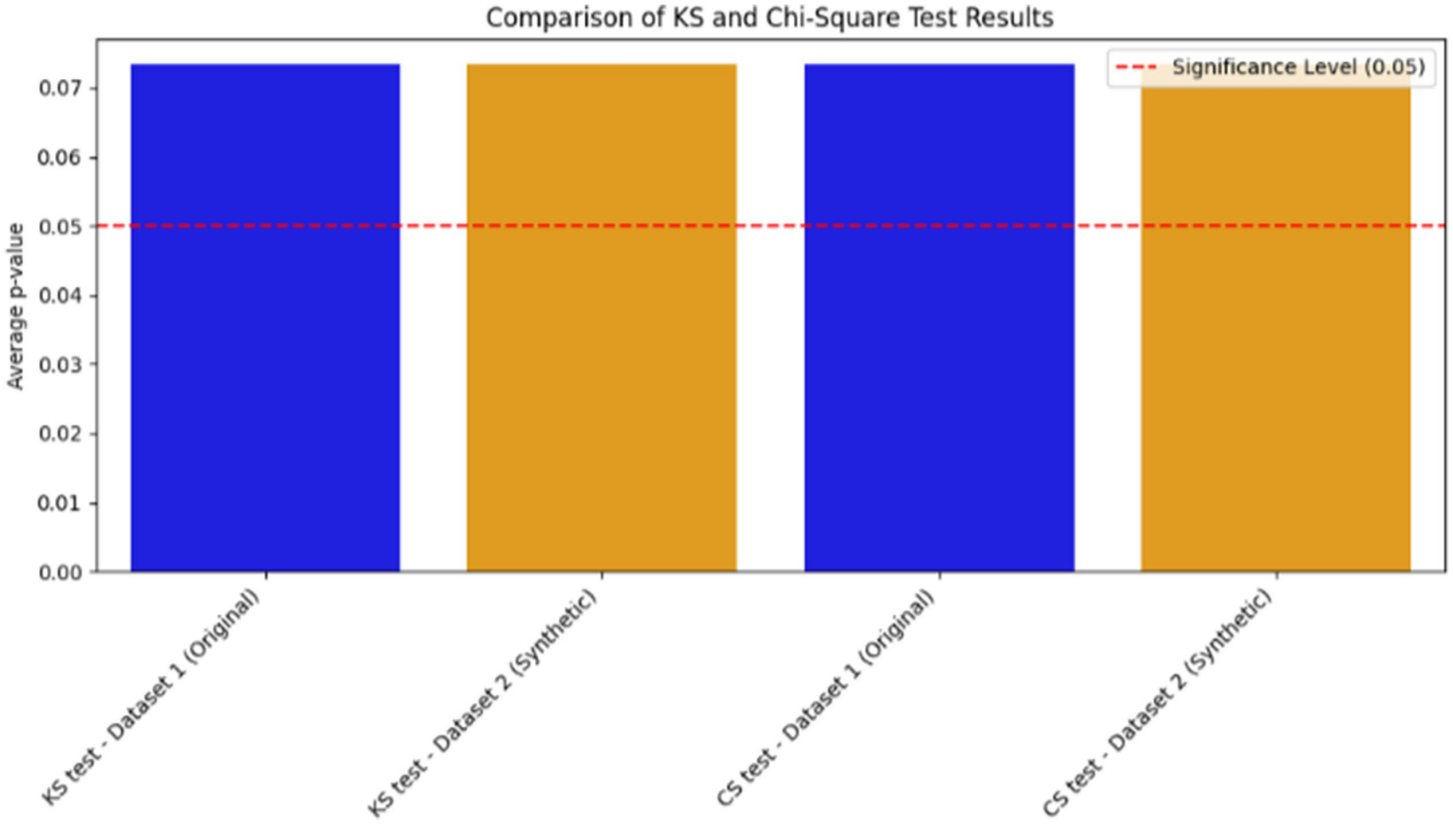
Evaluation of synthetic data quality with standard statistical metrics.

Furthermore, to evaluate the statistical distribution of numerical features and frequency distribution of categorical features on the synthetic data, standardized statistical metrics such as the Kolmogorov–Smirnov test (KS) and Chi-Square (CS) tests were performed. The KS test is used to validate whether the synthetic and original data’s numerical features preserve the same distribution. CS tests also assess the association of nominal features on both synthetic and original datasets. In the evaluation of the quality of the synthetic data using statistical tests such as KS and CS tests, the *p* value should be above the statistical significance cut-off (0.05), as indicated in most theories of statistical tests, to ensure a similar distribution of synthetic and original data(Andrade, 2019). Hence, the p value for our dataset’s statistical tests indicated in Figure 5 validates that the synthetic datasets are almost similar to the original datasets.

### 4.2 Performance evaluation metrics

**Figure 6:**
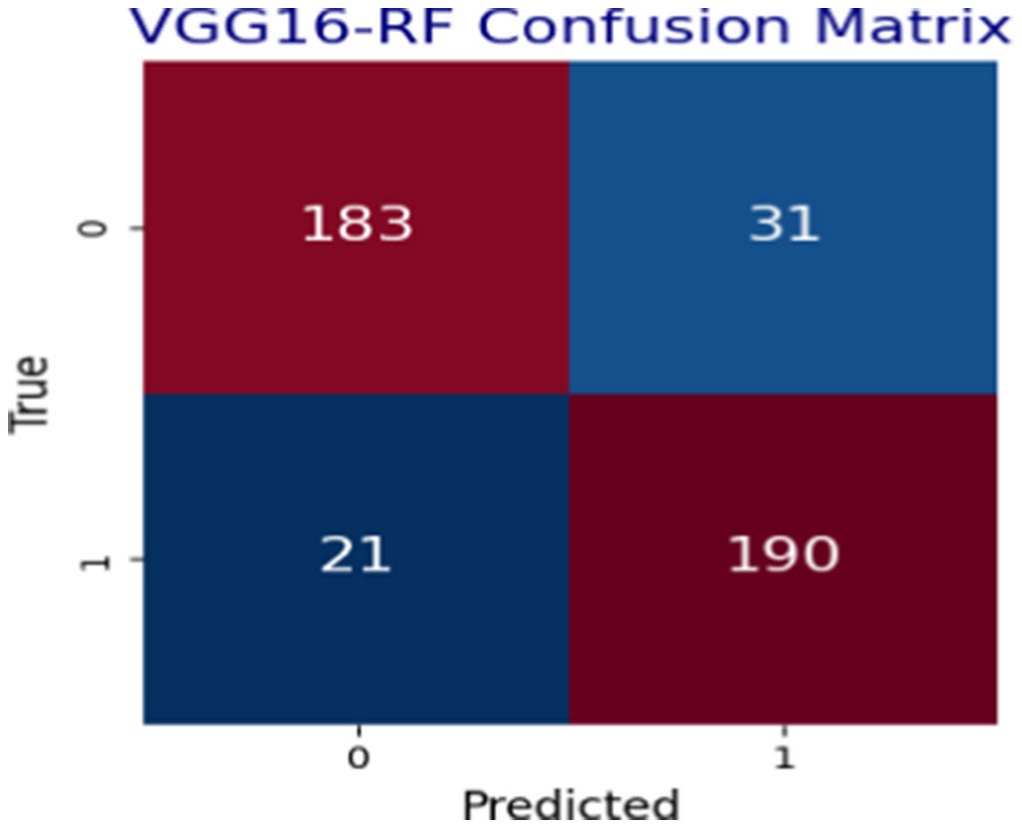
VGG16-RF model confusion matrix result.

The dataset was assessed using six hybrid classification algorithms, which were compared through 5-fold cross-validation to identify the most effective approach based on various statistical metrics, including accuracy. The algorithm evaluated includes VGG16-SVM, VGG16-GB, VGG16-DT, VGG16-LR, VGG16-RF, and VGG16-KNN. Different performance metrics were employed to measure the effectiveness of each hybrid approach. Various statistical measurement methods have been examined to determine the performance of different hybrid models. Accuracy, precision, recall, F-measure, sensitivity, and specificity were the main evaluation metrics utilized in this study. The predicted values generated during the testing processes, such as True Positive (TP), True Negative (TN), False Positive (FP), and False Negative (FN), are utilized to determine the results of evaluation metrics, as shown in Figure 6. Figure 6 shows the confusion matrix result of the proposed model over the test dataset. Detailed description of those evaluation metrics used to evaluate the performances of the models discussed as follow in Equations 3–8:

1. Accuracy: It represents the ratio of data points accurately predicted out of the total number of data points, expressed as the number of correctly predicted instances.

(3) $$\mathrm{Accuracy}=\frac{TP+TN}{TP+TN+FP+FN}$$

1. Precision: is the proportion of correctly predicted positive observations out of all the positive predictions made (Chandrasekhar and Peddakrishna, 2023).

(4) $$\mathrm{Precision}=\frac{TP}{TP+FP}$$

1. Recall: It is the proportion of instances correctly predicted as positive that actually belong to the positive class (Haq et al., 2020).

(5) $$\mathrm{Recall}=\frac{TP}{TP+FN}$$

1. F1 score is often called the F-measure, and it is the weighted harmonic mean of both precision and recall (Biswas and Samanta, 2021).

(6) $$F1-\mathrm{score}=2X\frac{\mathrm{Precision}\;X\;\mathrm{Recall}}{\mathrm{Precision}+\mathrm{Recall}}$$

1. Sensitivity is the proportion of actual positive samples correctly identified as positive in the test.

(7) $$\mathrm{Sensitivity}=\frac{TP}{TP+FN}$$

1. Specificity is the ratio of the number of true negative samples to the total number of samples that were tested as negative.

(8) $$\mathrm{Specificity}=\frac{TN}{FP+TN}$$

In Figure 6, the performance of the hybrid VGG16-RF is given with confusion matrices, which indicate the capability of the model in predicting heart disease situations using cleaned data in healthy or infected classes. The terms true positive (TP), true negative (TN), false positive (FP), and false negative (FN) are used to incorporate different evaluation metrics over the models analyzed in this study. Hence, both TP and TN terms represent correctly classified samples, while FP and FN refer to cases where the models made incorrect predictions. Figure 6 shows the confusion matrix result of the proposed model over the test dataset using actual and predicted values.

### 4.3 Experimental results

**Figure 7:**
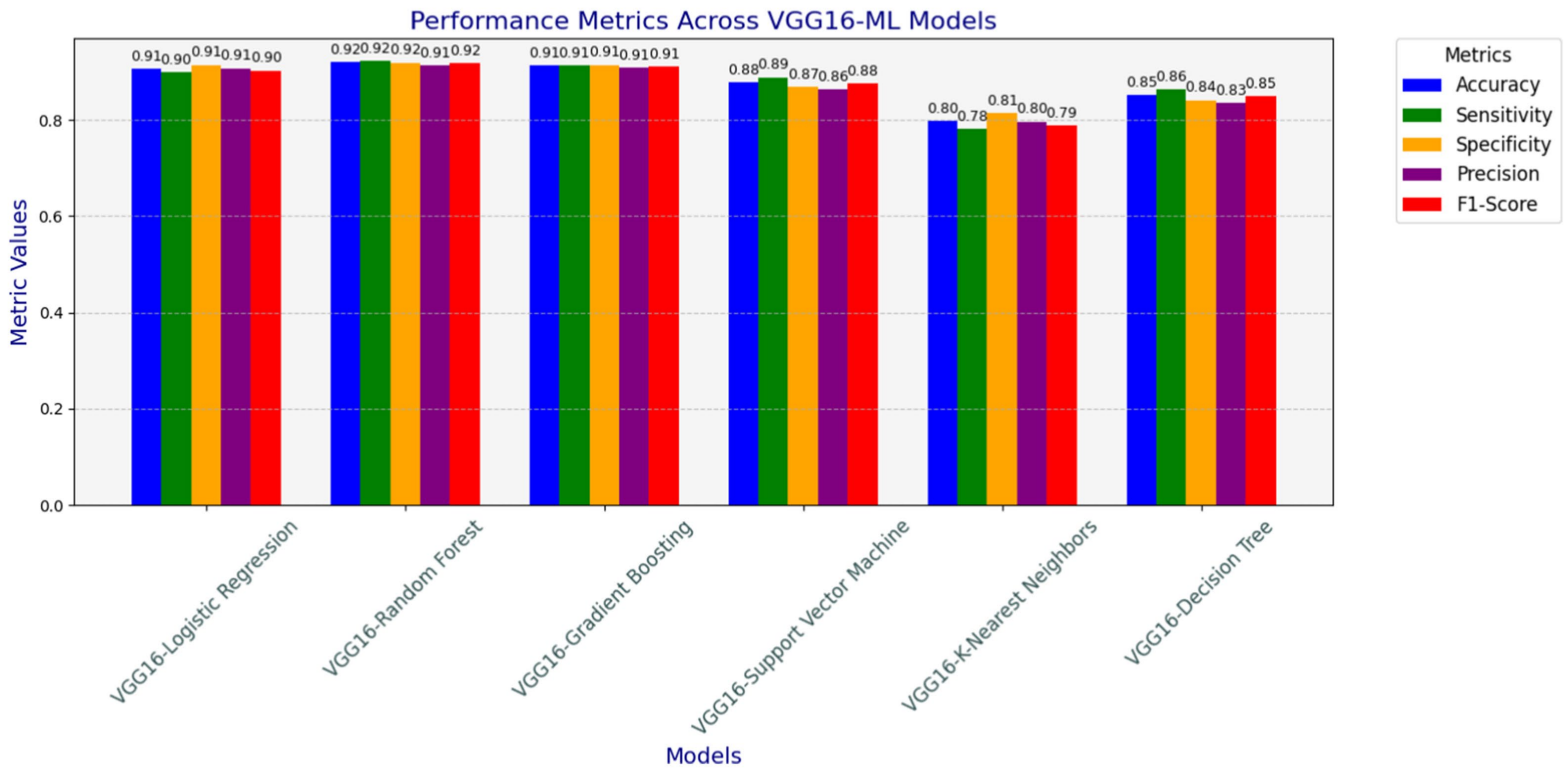
Accuracy, precision, recall (sensitivity), F1-score, and specificity results of hybrid models.

As depicted in Figure 7, different models have been examined for heart disease detection tasks by combining the strengths of both machine learning and deep learning approaches to build the hybrid model. The analyzed models in terms of accuracy, precision, recall, F1-score, and specificity are shown in Figure 7. The hybrid models, such as VGG16-GB, VGG16-SVM, VGG16-DT, VGG16-LR, VGG16-RF, and VGG16-KNN, have been applied using 5-fold cross-validation techniques on the preprocessed datasets used for this study. This section elucidates the classification performance report observed from these various hybridization techniques. The results show the overall performance of the six models, including accuracy, precision, recall, F1-score, sensitivity, and specificity, achieved during the analysis of the test datasets.

### 4.4 Comparative evaluation results of models

Table 2 shows the examined model’s accuracy result with the respective best parameters used during the hyperparameter tuning process. The datasets were split into five folds to apply a K-fold cross-validation testing mechanism to test the models using test data splits. Comparative evaluation results of the model’s accuracy result, with respective best parameters, are presented in Table 2. Additionally, Table 3 presents the confusion matrix results, which show the effectiveness of different hybrid models in detecting heart disease cases in normal or infected classes in terms of TP, TN, FP, and FN with their respective accuracy results.

**Table 2:** Best parameters and respective accuracy results of different models.

Models	Best parameters	Accuracy result
VGG16-Random Forest	max_depth = None, n_estimators = 50	92.00%
VGG16-Logistic Regression	C = 1, solver = liblinear	90.59%
VGG16-Decision Tree	max_depth = None, min_samples_split = 2	85.18%
VGG16-KNN	n_neighbors = 5, weights = uniform	79.76%
VGG16-Gradient Boosting	learning_rate = 0.1, n_estimators = 50	91.29%
VGG16-SVM	C = 0.1, and kernel = linear	87.76%

**Table 3:** Confusion matrix results of hybrid models.

Hybrid models	TP	TN	FP	FN
VGG16-RF	183	190	31	21
VGG16-LR	190	181	24	30
VGG16-DT	157	188	57	23
VGG16-SVM	183	178	31	33
VGG16-GB	171	188	43	23
VGG16-KNN	173	178	41	33

### 4.5 Testing the proposed models with unseen datasets

**Figure 8:**
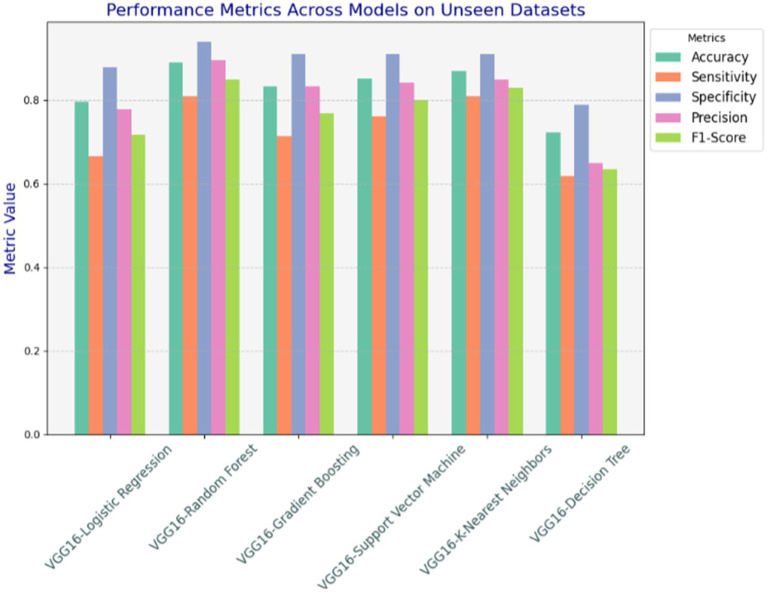
Performances of the proposed models with unseen datasets.

Conducting test mechanisms with unseen datasets was essential to assess the generalization capability of models after training the hybrid VGG16-ML models on training datasets. It was crucial to assess their performance on an entirely new dataset to evaluate generalizability and robustness. For this purpose, we tested the saved hybrid models on the Statlog heart disease dataset, which is obtained from the Kaggle website. The unseen Statlog dataset contains 270 samples of heart datasets and is slightly different compared to the initial training dataset. To ensure consistency, we applied preprocessing steps that mirrored those used during training, such as carefully handling missing values, feature encoding, outlier detection, normalization, and tabular data conversion. All models saved during the training are tested with the unseen datasets, and as indicated in Figure 8, the proposed hybrid VGG16-RF performs well and looks suitable for real-world deployment (Table 4).

**Table 4:** Performances of VGG16-ML models over unseen Statlog datasets.

Models	Accuracy	Sensitivity	Specificity	Precision	F1-score
VGG16-RF	88.89	80.95	93.94	89.47	85.00
VGG16-LR	79.63	66.67	87.88	77.78	71.79
VGG16-GB	83.33	71.43	90.91	83.33	76.92
VGG16-SVM	85.19	76.19	90.91	84.21	80.0
VGG16-DT	72.22	61.90	78.79	65.00	63.41
VGG16-KNN	87.04	80.95	90.91	85.00	82.93

As depicted in Table 5, the VGG16-RF model exhibited strong performance accuracy, sensitivity, specificity, precision, and F1-score on the unseen data sets, with minor drops in accuracy and sensitivity. However, its high specificity on the Statlog dataset demonstrates the model’s ability to generalize reasonably well to new data. This stability could be attributed to RF’s ensemble approach, which provides robustness to data variations.

**Table 5:** Comparisons of performances of VGG16-ML approaches against standalone Ml classifiers without VGG16.

Models	Evaluation metrics	Hybrid VGG16-ML	Without VGG16
SVM	Accuracy	87.76	58.35
	Recall	88.78	81.43
	Specificity	86.82	35.81
	Precision	86.26	55.34
	F1-Score	87.50	65.90
Random Forest	Accuracy	92.00	90.59
	Recall	92.20	90.48
	Specificity	91.82	90.70
	Precision	91.30	90.48
	F1-Score	91.75	90.48
Gradient Boosting	Accuracy	91.29	86.82
	Recall	91.22	86.67
	Specificity	91.36	86.98
	Precision	90.78	86.67
	F1-Score	91.00	86.67
Logistic Regression	Accuracy	90.59	79.06
	Recall	89.76	77.62
	Specificity	91.36	80.47
	Precision	90.64	79.51
	F1-Score	90.20	78.55
KNN	Accuracy	79.76	73.88
	Recall	78.05	71.43
	Specificity	81.36	76.28
	Precision	79.60	74.63
	F1-Score	78.82	72.99
Decision Tree	Accuracy	85.18	85.65
	Recall	86.34	83.81
	Specificity	84.09	87.44
	Precision	83.49	86.70
	F1-Score	84.89	85.23

## 5 Discussions

### 5.1 Interpretation of the result

The main objective of this study was to address the effectiveness of the transfer learning approach on machine learning techniques such as SVM, GB, RF, DT, KNN, and LR in heart disease detection processes. The driving point here was to integrate the feature extraction capabilities of pre-trained convolutional neural networks with machine learning techniques and verify which hybridization method will achieve better classification accuracy in heart disease predictions. As findings, promising results have been achieved by building a hybrid model of the VGG16 feature extractor with classical machine learning classification techniques for the heart disease prediction domain. The result achieved by the hybrid approach in terms of accuracy, precision, recall, F1-score, and specificity is discussed as follows.

The VGG16-SVM model performs promisingly in terms of an accuracy of 87.76%, sensitivity of 88.78%, specificity of 86.82%, precision of 86.26%, and an F1-score of 87.50%. These results indicate that VGG16-SVM, while performing reasonably well, is somewhat limited by slightly lower specificity and precision than ensemble methods. The use of VGG16 features enables the SVM classifier to classify with a high degree of sensitivity, indicating that it effectively identifies positive cases (cases with heart disease). However, its specificity and precision suggest it may be prone to misclassifying some negative cases as positive. Adjustments to the train VGG16-SVM with a much larger data size and applying regularization parameters may further optimize its performance.

The VGG16-GB achieves a strong balance between accuracy, recall, and specificity, making it an ideal model for heart disease detection, especially when compared to simpler models like DT or KNN. Gradient Boosting performed relatively well in heart disease detection, achieving an accuracy of 91.29, recall of 91.22, specificity of 91.36, precision of 90.78, and an F1-score of 91.00. VGG16-GB’s metrics demonstrate that it is well-suited for heart disease detection, particularly given its high accuracy and balanced sensitivity and specificity. It is a sequential approach to model improvement, where each tree corrects the errors of its predecessor, allowing VGG16-GB to capture subtle data patterns and enhance predictive accuracy. The balance between sensitivity and specificity further suggests that this model is capable of minimizing both false negatives and false positives, which is beneficial for the early detection and accurate diagnosis of heart disease. Its robustness in minimizing false positives and negatives, coupled with techniques like SHAP for interpretability, makes it a valuable model in medical applications like heart disease detection.

The VGG16-RF classifier achieved relatively promising scores of accuracy of 92.00%, sensitivity of 92.20%, specificity of 91.82%, precision of 91.30%, and an F1-score of 91.75%. These results highlight the VGG16-RF model’s robustness in heart disease detection. The high sensitivity and specificity scores indicate the model’s capability to reduce both false positives and false negatives effectively, which is critical in clinical decision-making. The ensemble nature of RF allows it to capture complex interactions within the dataset, and the use of multiple decision trees contributes to its resilience against overfitting. This performance underscores the suitability of RF in medical applications where predictive accuracy and reliability are paramount. The depth of the trees was not restricted (max_depth = None), allowing the model to fully explore the data patterns. The random forest classifier is beneficial for heart disease detection due to its ability to process both continuous and categorical data, effectively model nonlinear patterns, and adjust hyperparameters to enhance performance (Mulyani et al., 2024). As an ensemble method, it merges multiple decision trees, which improves its accuracy and robustness, making it a powerful tool for complex medical data.

The VGG16-LR also delivers an accuracy of 90.59%, a sensitivity of 89.76%, a specificity of 91.36%, a precision of 90.64%, and an F1-score of 90.20%. These metrics indicate that VGG16-LR performs well, particularly in maintaining a balance between sensitivity and specificity. The model’s linear nature aligns well with the features extracted by VGG16, suggesting that the processed data possesses a degree of linear separability that LR can effectively capture. The near-equal values of sensitivity and specificity imply that the model can accurately identify both positive and negative cases, making it a reliable choice for diagnostic purposes in medical settings. The fact that it achieved metrics identical to those of more complex models demonstrates that the core information is already well-captured by the preprocessing pipeline.

The VGG16-DT achieved an accuracy of 85.18%, a sensitivity of 86.34%, a specificity of 84.09%, a precision of 83.49%, and an F1-score of 84.89%. The VGG16-DT model provides moderate performance, with balanced sensitivity and specificity but somewhat lower precision. The metrics indicate that the DT model captures basic patterns within the data but may be prone to overfitting, especially when left unrestricted in depth. This can lead to reduced generalization, as evidenced by the relatively lower specificity. Decision trees tend to overfit when left unrestricted (max_depth = None), and their performance on unseen data can degrade. In practice, tuning for depth and regularization is critical to avoid overfitting.

The VGG16-KNN’s performance is significantly lower than that of the other models, with an accuracy of 79.76. Moreover, VGG16-KNN’s metrics suggest that its performance is somewhat limited in this application. The model’s lower sensitivity and specificity indicate challenges in maintaining high predictive accuracy, particularly in distinguishing between positive and negative cases. KNN’s reliance on instance-based learning may be less effective in the high-dimensional feature space derived from VGG16, which could explain the reduced accuracy and F1 score. While KNN may offer simplicity, its performance in this setting indicates it may not be ideal for heart disease detection, where reliable classification is essential.

### 5.2 Comparisons of hybrid approaches with standalone ML methods without VGG16

An experimental analysis was conducted by comparing the effectiveness of hybrid approaches with traditional machine learning methods without the VGG16 feature extraction processes by utilizing similar datasets. The hybrid approach is especially useful for models that rely on more complex feature representations, validating the effectiveness of transfer learning in heart disease detection. The hybrid models outperform their respective standalone traditional models, especially SVM and logistic regression models. Even though the random forest and decision tree stand-alone models achieve good performances, the hybrid model version exhibits even higher consistency in recall, precision, and F1-score. KNN shows a slight drop in performance when using VGG16, indicating that not all models benefit equally from feature extraction through the VGG16 pre-trained model. On the other hand, the integration of VGG16 feature extraction as a complementary addition enhances the standalone classifier’s performance, as demonstrated by SVM.

**Figure 9:**
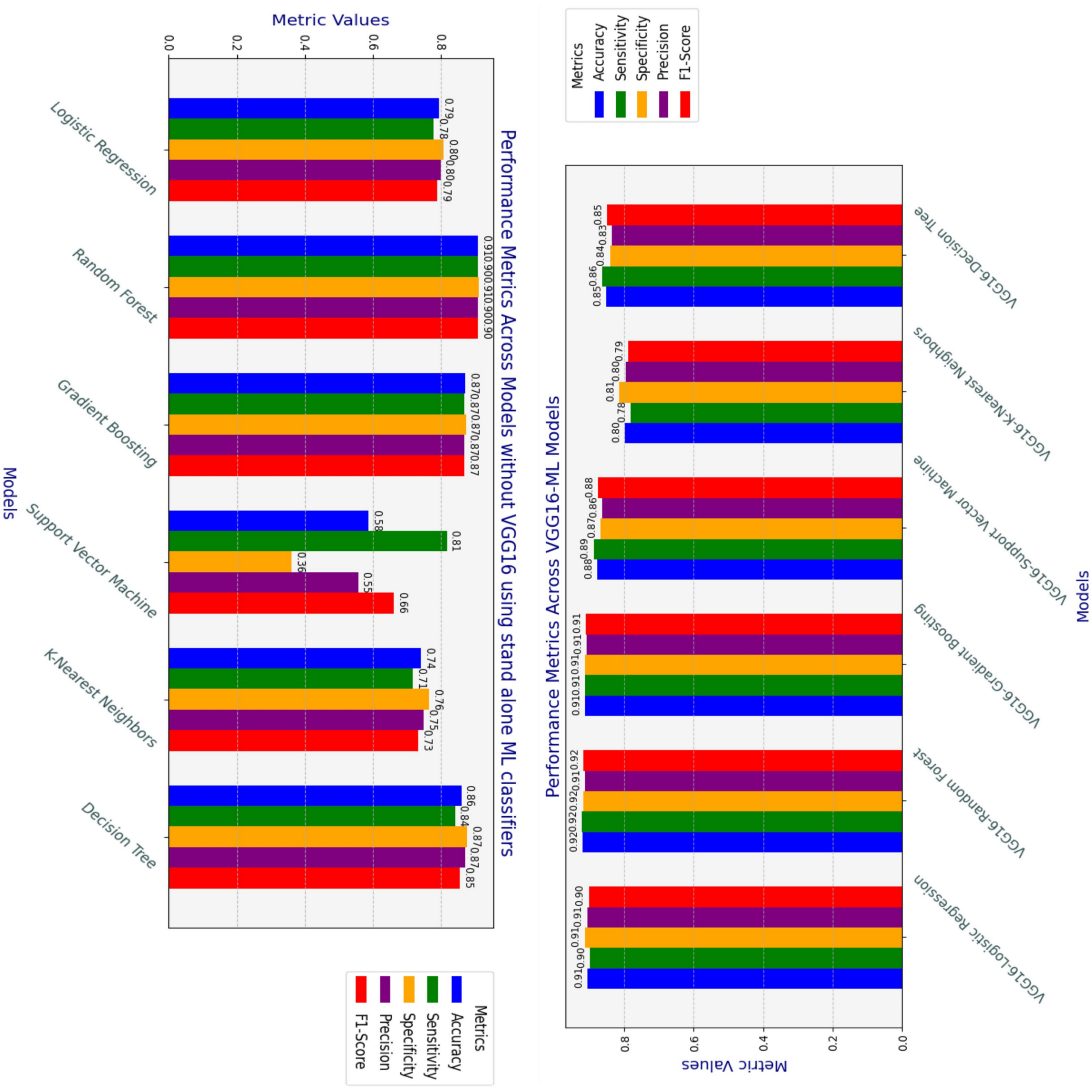
Comparison of Hybrid VGG16-ML and standalone classifiers performances.

As presented in Figure 9 and Table 5, the VGG16-ML hybrid approach provides a considerable boost in performance, especially in models that are more sensitive to feature extraction, such as SVM and Logistic Regression. On the other hand, random forests and decision trees perform well with or without VGG16. Overall, the findings of this study demonstrate that combining complementary techniques with the classical classification strengths of traditional machine learning models can effectively address the key challenges encountered in the heart disease detection domain.

### 5.3 Comparison of model complexity with standalone ML classifiers

**Figure 10:**
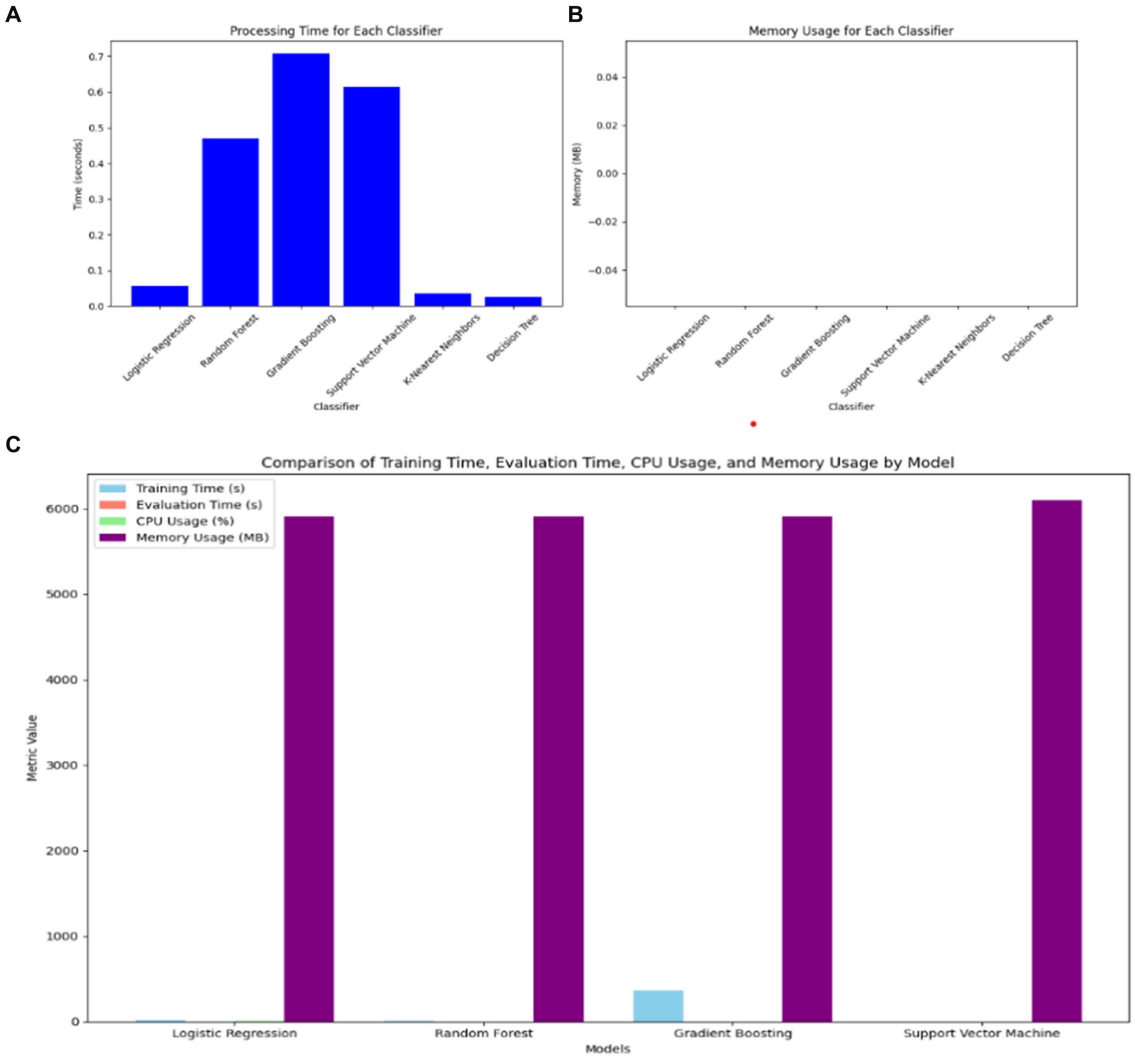
Comparison of VGG16-ML with individual classifiers’ complexity. **(A)** Processing time of ML classifiers. **(B)** Memory usage of ML classifiers. **(C)** Training time, evaluation time, CPU usage, and memory usage of VGG16-ML approaches.

In comparing the hybrid VGG16-ML models with standalone classifiers, the hybrid approach involving VGG16 typically exhibits a higher computational cost in terms of both processing time and memory usage. VGG16, as a deep learning model, requires significant resources for feature extraction, leading to increased processing times compared to standalone classifiers without feature extraction, as shown in Figure 10. Standalone models like DT, LR, and KNN demonstrate minimal processing times and memory demands, making them computationally efficient but potentially less effective in capturing complex patterns.

However, the hybrid VGG16-ML models justify their higher resource requirements by leveraging deep feature extraction to improve classification performance. By utilizing VGG16, the hybrid models gain a richer feature representation, which enhances their predictive power, particularly in complex, high-dimensional data scenarios like image-based or structured medical data. This makes the hybrid approach more suitable for applications where accuracy and interpretability supported by VGG16’s extracted features and XAI methods like SHAP are prioritized over computational speed. Thus, the trade-off between computational cost and predictive capability underlines the value of the hybrid VGG16-ML models, particularly for healthcare decision support systems where accuracy can outweigh the need for faster processing.

### 5.4 Comparison with the state-of-the-art

Heart disease detection using supervised machine learning methods is a well-explored field of research. Despite some unresolved challenges in this area, significant progress has been made with promising outcomes. In recent times, the application of hybrid deep learning-machine learning techniques to leverage their complementary strengths for heart disease detection has gained popularity. This study employed deep learning techniques for feature extraction from non-image data, integrating them with machine learning classifiers to predict heart conditions through transfer learning approaches. The research introduces novel methodologies, and the promising and directive results obtained are compared with state-of-the-art methods, as demonstrated in Table 6.

**Table 6:** Comparison of the proposed approaches with the state-of-the-art.

Study	Datasets	Methodology	Accuracy with unseen datasets	Hybridize with ML	Accuracy (in %)	Precision (in %)	Recall (in %)	F1-score (in %)	Specificity (in %)
Anbazhagan et al. (2024)	Tabular	XGBoost-LSTM	Undetermined	Yes	93.4	91	89.4	92	90
Mulyani et al. (2024)	Tabular	CNN end-to-end	Undetermined	No	100	100	100	100	Undetermined
Hossain et al. (2023)	Tabular	CNN-LSTM	Undetermined	No	74.15	81.82	72.07	76.62	77.11
Shrivastava et al. (2023)	Tabular	CNN-BiLSTM	Undetermined	No	96.66	96.84	96.66	96.63	Undetermined
The proposed method	Tabular	VGG16-RF	89%	Yes	92.00	90.64	89.76	90.20	91.82

The proposed hybrid VGG16-machine learning approach addresses key limitations observed in existing studies investigated for early prediction of heart disease by employing a hybrid of deep learning-machine learning approaches, including the study by Anbazhagan et al. (2024). In terms of data size, our study significantly expands the dataset size by employing CTGAN techniques, with 1,025 to 2,125 observations, to enhance the robustness and generalizability capability of the model. In contrast, the (Anbazhagan et al., 2024) model is limited to analyzing 918 samples only, which may restrict its applicability to diverse populations. A critical distinction of the proposed approach is also its evaluation of unseen datasets, using the publicly available Statlog dataset to assess its generalization capability. The results achieved during the test of the proposed model with these new and slightly different datasets confirm the model’s ability to maintain high predictive accuracy, generalizability capability, and reliability across different data distributions, underscoring its robustness in real-world scenarios. Such evaluations have not yet been employed on the recently conducted integrating machine learning-deep learning hybrid approaches for heart disease prediction AI-based solutions.

Our proposed hybrid of the VGG16-RF approach reshapes tabular data into an image-like format, enabling effective feature extraction using VGG16. This innovative adaptation demonstrates the versatility of transfer learning models in handling non-image data. Moreover, the combination of VGG16-extracted features with traditional machine learning classifiers, such as Random Forest, achieves competitive performance metrics, including 92% accuracy, 91.3% precision, and 92.2% recall. These results are comparable to or exceed those of the (Anbazhagan et al., 2024) model, which reported an accuracy of 93.4%, a sensitivity of 89.4%, and a precision of 91%. XAI is a critical aspect of similar models that have been developed recently. In a study by Anbazhagan et al. (2024), the model relies on the inherent interpretability of XGBoost. Our study integrates SHAP values as an XAI component to provide granular insights into feature importance. This ensures transparency in model predictions, facilitating trust and acceptance among healthcare professionals.

Furthermore, the use of CTGAN in this proposed approach for data augmentation demonstrates the scalability of our approach, enabling its application in different domains where the size of datasets is an issue. Such techniques bring contributions that are particularly relevant in medical research areas, where obtaining large observations and annotated datasets is often challenging. By addressing these limitations and incorporating rigorous evaluation of unseen datasets, the proposed hybrid VGG16-machine Learning approach represents a significant advancement in AI-driven heart disease detection.

The hybridization of VGG16’s feature extraction capabilities with supervised machine learning classifiers offers a powerful approach to handling non-image data in predictive tasks like heart disease detection. This method leverages the strengths of both deep learning and traditional machine learning techniques, creating a model that benefits from the advanced feature extraction capabilities of VGG16 and the robust classification performance of supervised algorithms like random forest, gradient boosting, and SVM. The integration of transfer learning allows pre-trained weights from image datasets to be applied to non-image data. This reduces the need for large datasets in medical applications, where data scarcity is often an issue. The pre-trained VGG16 model serves as an efficient feature extractor, reducing training time while improving feature quality.

Generally, hybridizing VGG16’s deep feature extraction capabilities with supervised machine learning classifiers results in an effective system for heart disease detection and similar tasks. The deep learning model’s ability to capture complex, high-level features, combined with the classification strength of traditional machine learning algorithms, leads to outstanding performance results. This approach offers a promising solution for predictive analytics in domains where data may be limited, but accuracy is paramount, such as medical diagnostics.

## 6 Conclusion

This study explored heart disease detection by integrating deep learning techniques with supervised machine learning classifiers through a transfer learning approach. The hybrid approach utilizing VGG16 for feature extraction and supervised classifiers such as SVM, gradient boosting, random forest, logistic regression, KNN, and decision tree was thoroughly evaluated. The findings indicate that the hybrid approach significantly surpasses traditional machine learning classifiers, particularly in heart disease predictions. Notably, the VGG16-Random Forest hybrid model delivered perfect performance across all metrics, with 100% accuracy, precision, recall, F1-score, and specificity. This is a substantial improvement compared to the performance of the same classifiers when used independently without deep learning-based feature extraction.

Limitations and future directions: Even though the proposed hybrid of the deep learning and machine learning approach with XAI achieves promising performance on heart disease detection, we acknowledge issues of *computational costs* (may pose challenges during deployment in resource-constrained settings), *limited evaluation of robustness* (robustness against noisy inputs was not explicitly analyzed), *XAI scope(*integration of hybrid argumentation-based XAI was not explored), and *data size and diversity*(larger data size and demographic as well as clinical diversity were not addressed) as potential limitations of this study.

The key insight observed in this study was that applying pre-trained models like VGG16’s deep feature extraction potentially enhances the performance of machine learning models, enabling them to better capture complex relationships in the data. The study highlights the effectiveness of combining deep learning feature extraction with machine learning classifiers through a transfer learning mechanism, especially in cases involving non-image data. The hybrid VGG16-ML models with suitable data conversion methods and hyperparameter tuning techniques provide a significant performance boost, allowing for highly accurate and reliable heart disease detection. These results emphasize the potential of hybrid models in medical diagnostics, suggesting that future research on hybrid argumentation-based and SHAP explanation approaches, using more diverse clinical populations and datasets to validate models’ generalization capability and optimize models’ computational costs, should continue to explore better integration of deep learning and machine learning techniques to address other complex prediction tasks in healthcare.

## Data Availability

Publicly available datasets were analyzed in this study. This data can be found at: https://www.kaggle.com/datasets/johnsmith88/heart-disease-dataset, Kaggle.
